# Multi-Omics Revealed the Protective Effects of Rhamnolipids in Lipopolysaccharide Challenged Broilers

**DOI:** 10.3389/fimmu.2022.824664

**Published:** 2022-02-18

**Authors:** Ruiqiang Zhang, Xueyan Shi, Yuqi Chen, Jinsong Liu, Yanping Wu, Yinglei Xu

**Affiliations:** ^1^ College of Animal Science and Technology, College of Veterinary Medicine, Zhejiang Agricultural and Forestry University, Hangzhou, China; ^2^ Institute of Animal Health Products, Zhejiang Vegamax Biotechnology Co., Ltd., Anji, China

**Keywords:** multi-omics, rhamnolipid, lipopolysaccharides, metabolome composition, microbiota, broiler

## Abstract

Rhamnolipid (RL) is a glycolipid biosurfactant and exhibits the following outstanding characteristics: strong antibacterial properties, low toxicity, and high biodegradability. The present research was conducted to explore the protective effects and mechanisms of rhamnolipids as an alternative to antibiotics in LPS (lipopolysaccharide)-challenged broilers. 16S rRNA gene sequencing and metabolomics were used for analyzing the cecal microbial composition and serum metabolites. Dietary antibiotics and RLS supplementation decreased the weight loss rate, enhanced serum immunoglobulin levels, reduced serum diamine oxidase and D-lactate acid concentration, and improved the symptoms of intestinal bleeding and villus height, when broilers were challenged with LPS. The addition of RLS in the diet enhanced serum interleukin-4 and interleukin-10 contents and reduced serum interleukin-6 and tumor necrosis factor-α levels in LPS-challenged broilers compared with the antibiotics group. Spearman’s correlation analysis revealed that RLS may alleviate LPS-induced inflammatory responses through altering the 6-methoxymellein level in broilers. The genus *Bacteroides* may contribute to the decreased weight loss rate *via* regulating the serum lysoPC [20:5(5Z,8Z,11Z,14Z,17Z)] secretion. RLS alleviates LPS-induced intestinal injury, enhances the growth and immunity, ameliorates intestinal microflora, and improves serum metabolites in LPS-challenged broilers. RLS exhibited better protective effect than antibiotic supplementation in the diet of LPS-challenged broilers. These findings provide potential regulation strategies and novel insights for RLS enhancing its protective effect in LPS-challenged broilers.

## Introduction

In the last decades, antibiotics were used as feed additives in food animals to improve the growth and feed utilization, prevent infectious diseases, and reduce morbidity and mortality (1). However, antibiotics used in feed have caused potential undesirable effects, such as food animal products antibiotic residues, environmental pollution, and the production of drug-resistant bacteria (2). To address this, the continuous administration of antibiotics used in animal commercial feed has been banned in China and the European Union (3, 4). Therefore, there is increasing attention for antibiotic alternatives in the food animal industry.

Rhamnolipids are versatile anionic glycolipid biosurfactants and mainly produced by various bacterial species including some *Pseudomonas* and *Burkholderia* strains (5). It has the outstanding characteristics of strong antibacterial properties, low toxicity, and high biodegradability, and is used in a wide range of fields such as medical, environmental, and food (6). Intestine is the largest immune organ and plays a vital role in digestion and absorption of nutrients (7). Intestinal mucosa represents the most vital line of defense against harmful substances, such as toxins, pathogens, and antigens, preventing them from entering the host (8). Our previous studies found that rhamnolipids (RLS) could improve the growth performance, enhance the immunity and intestine histology, and optimize the cecal microflora, and the opportune level in the diet of birds is 1,000 mg/kg (9, 10).

The endotoxin lipopolysaccharide (LPS) is present in the outer membrane of Gram-negative bacteria (11). It has been extensively used as a model for immunological stress in laboratory animals. Abdominally injected LPS can induce oxidative stress, which then leads to the production of reactive oxygen species, and, in turn, causes inflammation, changes the distribution of nutrients, decreases the growth potential, induces various diseases, and even leads to death, in mouse (12), pig (13), and broiler (14). For all we know, limited research investigating the supplementation of RLS as an alternative to antibiotics alleviates lipopolysaccharide-induced inflammatory responses in broilers.

Metabolomics can measure small-molecule metabolites, which can give important knowledge of the components of various clinical conditions, and has unique advantages in facilitating the understanding of physiological and pathological characteristics in biological samples (15, 16).

The present study was performed to evaluate the application of RLS as an alternative to antibiotics in broilers diet effects on immunity, intestinal function, microbiota, and metabolome composition in response to LPS challenge through an untargeted metabolomics analysis and 16S rRNA gene sequencing approach. Results of the combined omics may help to identify the biomarker metabolites, as well as illuminate the underlying microbiological mechanism in LPS-challenged broilers supplemented with RLS.

## Materials and Methods

### Animals and Treatment

A total of 48 one-day-old male Ross 308 broilers with similar body weight (average initial body weight: 38.98 ± 0.16) were randomly assigned to 4 treatment groups with 12 birds per treatment. The treatments were as follows: (1) control group: birds fed a basal diet + saline challenged; (2) LPS group: birds fed the basal diet + LPS challenged; (3) antibiotics group: birds fed the basal diet plus 75 mg/kg chlortetracycline + LPS challenged; (4) RLS group: birds fed the basal diet plus 1,000 mg/kg RLS + LPS challenged. The basal diet (**Table S1**) was formulated as per the nutrient requirements of broilers as recommended by the National Research Council (1994). The LPS was obtained from *Escherichia coli* (L2880, Sigma, USA) and dissolved in 154 mmol/L sterile saline solution with 1 mg/mL. The birds were administered an intraperitoneal injection of LPS at a dose of 1 mg/kg body weight or the same amount of sterile saline solution at 8:00 a.m. on days 42, 43, and 44, respectively. All broilers were placed in an environmentally controlled lab and allowed *ad libitum* access to water and mash feed.

### Sample Collection

Three hours after the last LPS injection at age 44 days, eight broilers from each group were randomly selected for sampling. Blood samples were obtained from the wing vein and then centrifuged at 4000×*g* for 10 min at 4°C. Subsequently, the serum samples were collected and stored at −80°C. Then, birds were euthanized *via* cervical dislocation. The jejunum and ileum were separated according to a previous study (17). The section of jejunum and ileum with a length of about 1.5 cm was sliced at the middle position and fixed in 10% cold formalin for histological analysis. The digesta samples of cecum were excised aseptically and rapidly removed to a sterile cryogenic vial and stored at −80°C for subsequent tests.

### Body Weight

The birds were weighed before and after challenge. The weight loss rate (WLR) was calculated using the following formula:


$$\text{WLR (\%)}=100\times \frac{\text{body weight before LPS challenge}-\text{body weight after LPS challenge}}{\text{body weight before LPS challenge}}$$

### Serum Biochemical Status

Detection kits were used to determine the serum biochemical parameters, IgA (immunoglobulin A), IgY (immunoglobulin Y), IgM (immunoglobulin M), IL-4 (interleukin-4), IL-6 (interleukin-6), IL-10 (interleukin-10), IL-1β (interleukin-1β), and TNF-α (tumor necrosis factor-α), by immunoturbidimetry according to the manufacturer’s instructions. The intestinal permeability biomarkers in serum, including D-lactate acid (D-LA) and diamine oxidase (DAO), were measured by the appropriate detection kits following the protocols of the manufacturers. The commercial kits were purchased from Nanjing Angle Gene Bioengineering Co., Ltd. (China)

### Intestinal Morphology Analysis

Intestinal fragments of the jejunum and ileum were dehydrated in gradient concentrations of ethanol solutions and cleared with xylol and then embedded in paraffin. Each fixed sample was sectioned into 5-µm thickness with a microtome and stained with hematoxylin–eosin (H&E) (18). The villus height and crypt depth were determined employing an inverted microscope, and the villus height/crypt depth ratios were calculated.

### Real-Time PCR Analysis

Total RNA from each mucosal sample was isolated with RNAiso reagent (Takara Bio, Inc., China). Total RNA quality and concentration were determined using a Nano-300 micro-spectrophotometer (Hangzhou Allsheng Co., Ltd., China). The RNA samples were diluted to 500 ng/µL with water (treated with diethyl pyrocarbonate) and then reverse transcribed using a commercial kit (RR047, Takara Bio, Inc., China). The primer sequences are displayed in **Table S2** and synthesized by Hangzhou TSINGKE Biological Technology (China). The Bio-Rad CFX96 Real-Time System and RR420A commercial RT-PCR kit (Takara Bio, Inc., China) were used to execute the PCR program. The cycle time values of target genes were analyzed to determine the expression levels using the 2^−ΔΔCT^ method and normalized to β-actin (19).

### Cecal Microbiota Composition

The microbial genomic DNA of samples were extracted utilizing a TIANamp Stool DNA kit (Tiangen Biotech Co., Ltd, China) following the protocols of the manufacturers. DNA purity and concentration were monitored on 1% agarose gels and then diluted to l μg/mL. The bacterial 16S rRNA gene V3–V4 hypervariable regions were amplified using the barcode with primers 338F (ACTCCTACGGGAGGCAGCAG) and 806R (GGACTACHVGGGTWTCTAAT). The 16S rRNA gene V3–V4 hypervariable regions were analyzed using PE300 Illumina MiSeq platform by Shanghai MajorBio Bio-Pharm Technology CO., Ltd. (China).

The FLASH1.2.1 operational taxonomic units (OTUs) were used to quality-filter the raw data through Trimmomatic and merged process. OTUs were clustered with 97% sequence similarity using UPARSE (version 7.1) with a novel “greedy” algorithm that does chimera filtering and OTU clustering simultaneously. The taxonomy of each 16S rRNA gene sequence was conducted using an RDP Classifier algorithm (http://rdp.cme.msu.edu/) against the Silva (SSU123) 16S rRNA database using a confidence threshold of 70%. The QIIME1.9.1 was used to perform the analysis of β diversity based on the unweighted UniFrac distance. The microbiota composition was analyzed based on the tax_summary and R package version 3.3.1, and data were measured by one-way ANOVA and Tukey’s test. Microbial functions were predicted by Greengenes-based PICRUSt and differential analysis was performed by the STAMP software.

### Cecal Volatile Fatty Acids

Approximately 1 g of cecal chyme sample was mixed with 6% phosphorous acid (m/v, 1:3). Then, the supernatant was separated following vibration and centrifugation protocols. The volatile fatty acids, including acetic acid, propanoic acid, isobutyric acid, butyric acid, isovaleric acid, and valeric acid, were detected using GC7890B gas chromatography with a column (122-3232, DB-FFAP, 30 m × 0.25 mm × 0.25 μm, Agilent Technologies, USA). The volatile fatty acid external standards were purchased from Sigma-Aldrich (Shanghai, China).

### Metabolome Composition

The analysis procedure of serum metabolites used in this study has been described previously (10). Briefly, the metabolites were extracted, homogenized, ultrasonicated, and placed to precipitate proteins. After centrifugation, an ACQUITY UPLC BEH C18 column (100 mm × 2.1 mm i.d., 1.7 µm; Waters, Milford, USA) equipped in the ExionLCTMAD system was used to analyze the supernatant samples. The UPLC system was coupled to a Q-TOF Mass Spectrometer (AB Sciex, USA), equipped with an electrospray ionization source operating in negative and positive ion mode. The mixing of all samples at an equal volume ratio was prepared and tested in the same manner for quality control.

The Progenesis QI 2.3 (Nonlinear Dynamics, Waters Corporation, Milford, MA, USA) was used to preprocess the raw data. The metabolic features detected in >80% of the samples were retained. Then, metabolites were identified by matching the exact molecular mass data (m/z) of the samples against METLIN (http://metlin.scripps.edu/) and the Human Metabolome Database (http://www.hmdb.ca/). Analysis for multivariate statistics was executed using the ropls (Version1.6.2, http://bioconductor.org/packages/release/bioc/html/ropls.html) package of R software from Bioconductor on a Majorbio cloud platform (https://cloud.majorbio.com). The potential metabolic biomarkers were screened with a critical VIP > 1.0, *p* < 0.05. Metabolites were clustered using hierarchical clustering and averaging. Pathway analysis and enrichment analysis based on the Kyoto Encyclopedia of Gene and Genomes database (http://www.genome.jp/kegg/) were performed to examine the possible biological roles of candidate metabolites.

### Statistical Analysis

Data in this research were preliminarily processed using Excel 2019. Then, Duncan’s multiple range test was used to compare the means of treatment at *p* < 0.05 significance levels through Prism software 8.0 (GraphPad Software Inc., United States) and SPSS 22.0 (SPSS Inc, USA). The results were exhibited as the mean and the standard errors of means.

## Results

### Body Weight

The body weight of broilers exhibited no difference among the group before and after LPS challenge (*p* > 0.05, **Table 1**). Compared with the control group, LPS-challenged birds had enhanced the weight loss rate (*p* < 0.05). Dietary antibiotics and RLS supplementation decreased the weight loss rate when birds were challenged with LPS (*p* < 0.05).

**Table 1:** Rhamnolipids attenuated the compromised growth in broilers challenged with lipopolysaccharide.

Items^1^	CON	LPS	ANT+LPS	RLS+LPS	SEM	*p*-value
IBW, g	2,445.38	2,509.45	2,514.25	2,579.88	34.35	0.611
FBW, g	2,287.5	2,166.25	2,248.75	2,331.25	34.29	0.386
WLR, %	6.39^c^	13.70^a^	10.63^b^	9.69^b^	14.14	<0.001

^abc^Means that do not share the same superscript in each row are significantly different, p < 0.05 (**Table 1**).

^1^IBW, average body weight before challenged; FBW, average body weight after challenged; WLR, Weight loss rate; CON, broilers fed a basal diet and challenged with sterile saline solution; LPS, broilers fed a basal diet and challenged lipopolysaccharide; ANT+LPS: broilers fed a basal diet supplemented with 75 mg/kg chlortetracycline and challenged with lipopolysaccharide; RLS+LPS: broilers fed a basal diet supplemented with 1,000 mg/kg rhamnolipids and challenged with lipopolysaccharide.

### Serum Biochemical Parameters

**Figure 1:**
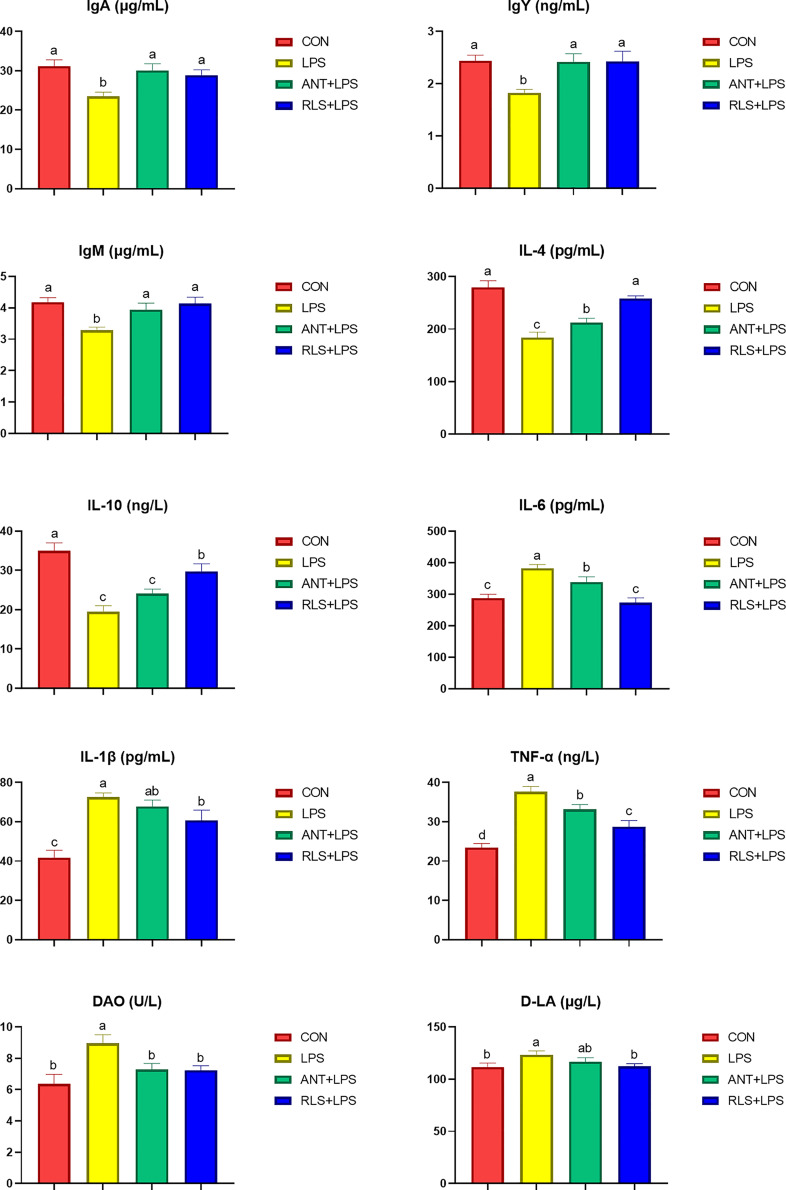
Rhamnolipids ameliorated the serum biochemical indexes in broilers challenged with lipopolysaccharide ^(abc^RE 2 | Rhamnolipids alleviated intestinal injury in broilers challenged with lipopolysaccharid means that do not share the same superscript are significantly different, *p* < 0.05.

LPS challenge reduced the levels of serum IgM, IgY, and IgA; decreased the serum IL-4 and IL-10 contents; enhanced serum IL-6, IL-1β, and TNF-α levels; and increased serum D-LA and DAO concentration compared with the control group in broilers (*p* < 0.05, **Figure 1**). Dietary antibiotics addition had higher serum IgA, IgY, IgM, and IL-4 contents, and lower serum IL-6, TNF-α, and DAO levels in broilers (*p* < 0.05). RLS inclusion exhibited increased IgM IgY, and IgA, levels; enhanced IL-4 and IL-10 contents; decreased IL-6, IL-1β, and TNF-α levels; and reduced D-LA and DAO concentration of serum in broilers (*p* < 0.05). The addition of RLS in the diet enhanced serum IL-4 and IL-10 contents and reduced serum IL-6 and TNF-α levels in broilers compared with the antibiotics group (*p* < 0.05).

### Intestinal Morphology

**Figure 2:**
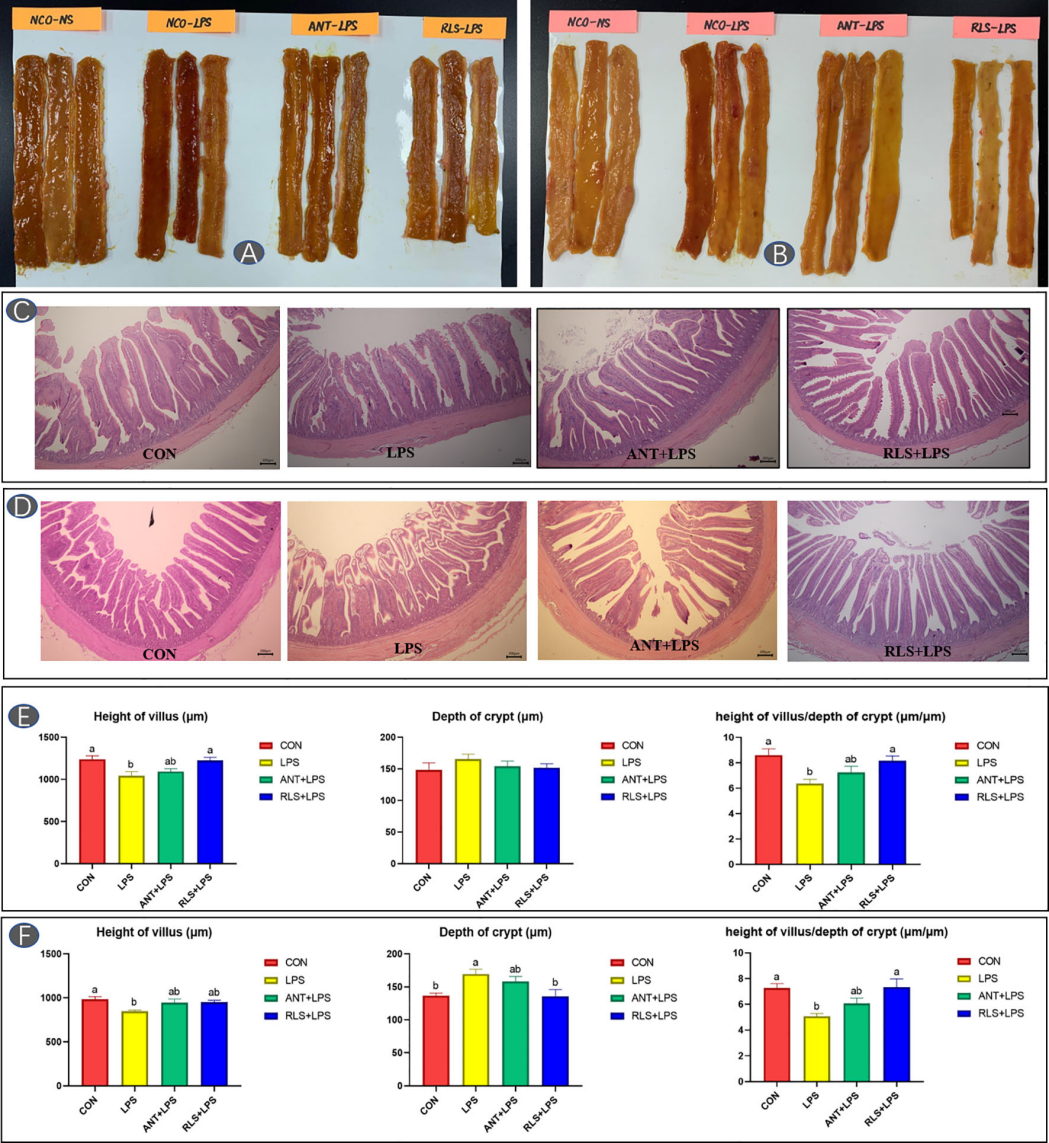
Rhamnolipids alleviated intestinal injury in broilers challenged with lipopolysaccharide (**A, C**, **E**: jejunum; **B, D**, **F**: ileum; ^ab^means that do not share the same superscript are significantly different, p < 0.05).

The status and morphology in jejunum and ileum of broilers are shown in **Figure 2**. Broilers in the LPS group displayed obvious bleeding point in jejunum and ileum. Antibiotics and RLS supplementation improved the symptoms of intestinal bleeding in broilers. LPS challenge reduced the villus height, decreased the ratio of villus height to crypt depth of jejunum and ileum, and increased the crypt depth of ileum in broilers (*p* < 0.05). The jejunum of birds in the RLS group had higher villus height than that of birds in the LPS group (*p* < 0.05). The ileum in the RLS group had increased the villus height-to-crypt depth ratio and decreased crypt depth compared to that of birds in the LPS group (*p* < 0.05).

### Intestinal Messenger RNA Expressions

**Figure 3:**
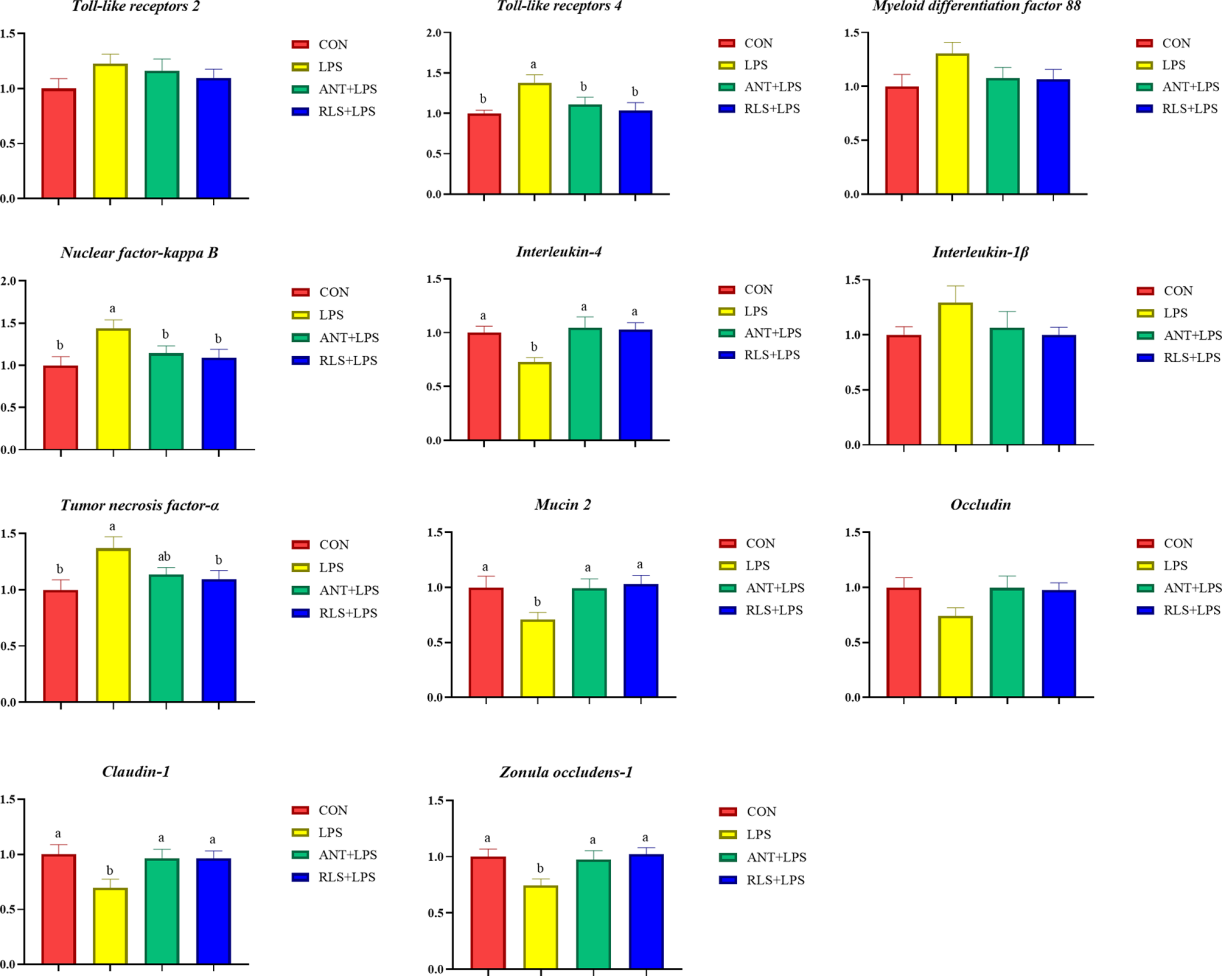
Rhamnolipids modulated the relative gene expression of jejunum mucosal barrier function in broilers challenged with lipopolysaccharide (^ab^means that do not share the same superscript are significantly different, p < 0.05).

**Figure 4:**
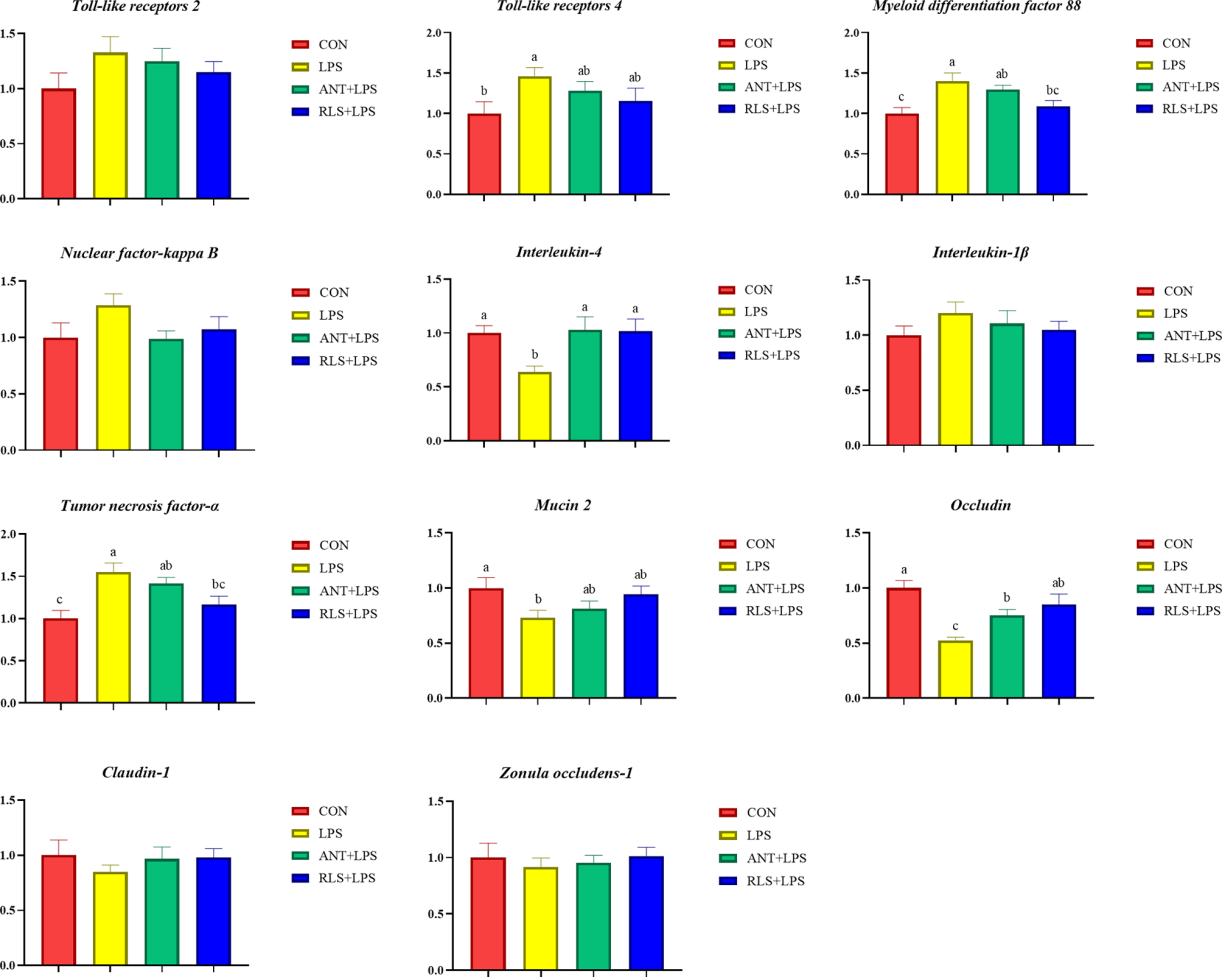
Rhamnolipids modulated the relative genes expressions of ileum mucosal barrier function in broilers challenged with lipopolysaccharide (^abc^means that do not share the same superscript are significantly different, p < 0.05).

LPS challenge upregulated *TLR 4*, *NF-κB*, and *TNF-α* expression levels and downregulated *IL4*, *MUC-2*, *Claudin-1*, and *ZO-1* expression levels of jejunum mucosa in broilers (*p* < 0.05, **Figure 3**). Compared with the LPS group, antibiotics inclusion reduced the expression levels of *TLR 4* and *NF-κB*, and enhanced the expression levels of *IL4*, *MUC-2*, *Claudin-1*, and *ZO-1* of jejunum mucosa in broilers (*p* < 0.05). RLS inclusion decreased the *TLR 4*, *NF-κB*, and *TNF-α* expression levels, and increased the *IL4*, *MUC-2*, *Claudin-1*, and *ZO-1* expression of jejunum mucosa in broilers. **Figure 4** shows the gene expression of ileum mucosa in broilers. LPS challenge upregulated the expression of *TLR 4*, *MyD88*, and *TNF-α*, and downregulated the expression levels of *IL 4*, *MUC-2*, and *Occludin* (*p* < 0.05). Compared with the LPS group, antibiotics inclusion upregulated *IL 4* and *Occludin* expression (*p* < 0.05). RLS inclusion downregulated the expression of *MyD88* and *TNF-α*, and upregulated the expression levels of *IL 4* and *Occludin* (*p* < 0.05). It has no significant difference on the expression levels of *TLR 2*, *TLR 4*, *MyD88*, *NF-κB*, *IL-4*, *IL-1β*, *TNF-α*, *MUC-2*, *Occludin*, *Claudin-1*, and *ZO-1* of jejunum and ileum mucosa in broilers between the antibiotics group and RLS group (*p* > 0.05).

### Cecum Microbiota

**Figure 5:**
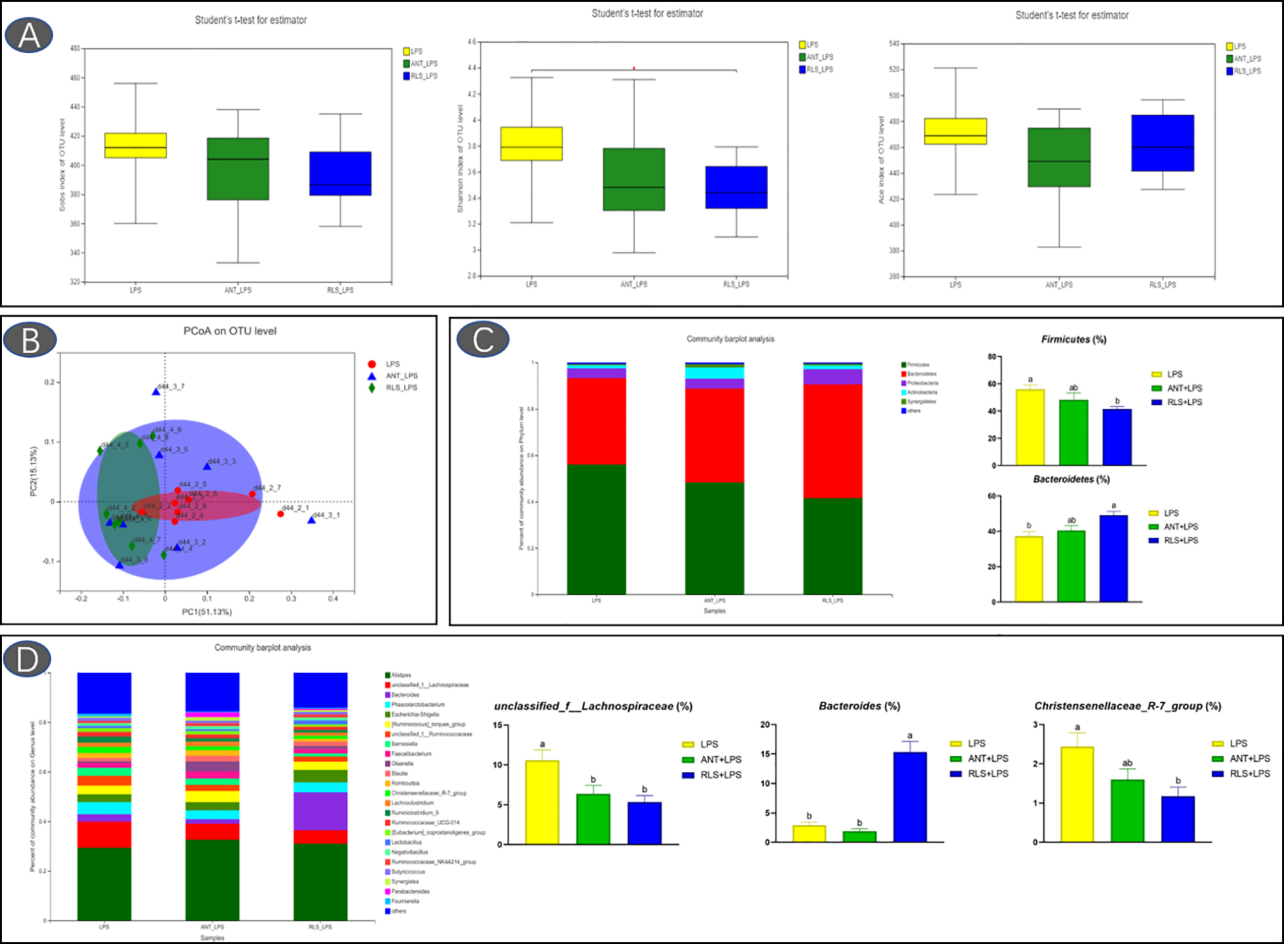
Rhamnolipids improved cecal microbiota diversity in broilers challenged with lipopolysaccharide based on OTU level. (**A**: the Sobs index, Shannon index, and ACE index reflect species diversity within and among groups; **B**: the principal co-ordinates analysis plot; **C**: microbial composition at the phylum level. **D**: microbial composition at the genus level; ^ab^means that do not share the same superscript are significantly different, p < 0.05).

**Figure 6:**
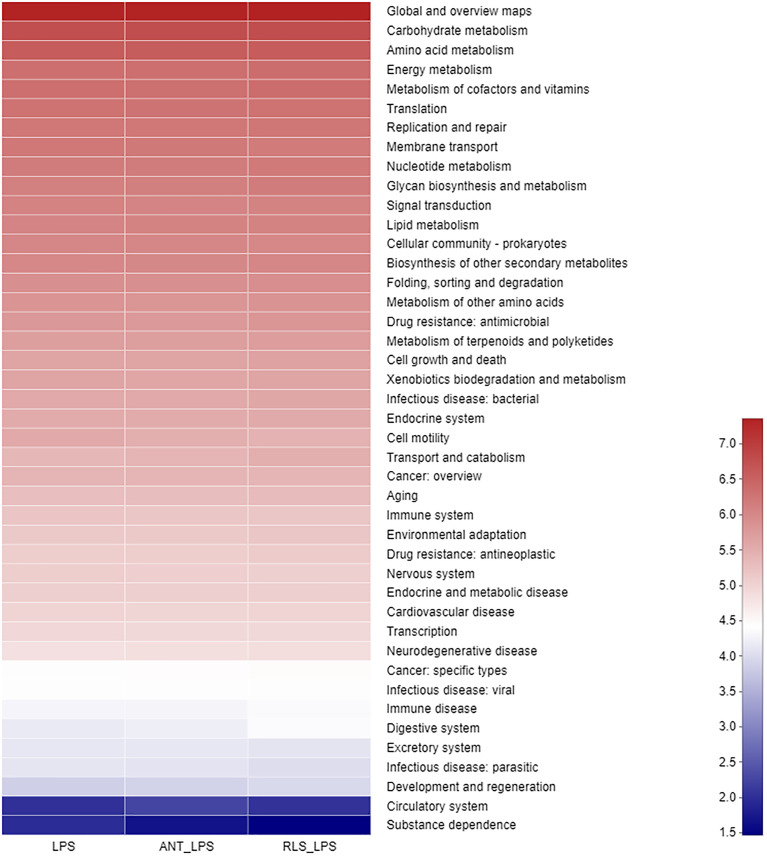
The PICRUSt pathway heatmap of cecal microbiota in broilers challenged with lipopolysaccharide.

The Shannon index of cecal microbiota in the RLS group exhibited a lower value than that of broilers in the control group (*p* < 0.05, **Figure 5**). The separated PCoA plot revealed that the microbiota composition of broilers in the LPS group is different from that in the RLS addition group. The taxonomic classification revealed that the dominant phyla are Firmicutes, Bacteroidetes, Proteobacteria, Actinobacteria, and Synergistetes in broilers. The reduced Firmicutes and increased Bacteroidetes were observed in broilers fed with the RLS inclusion diet (*p* < 0.05). The *Alistipes*, *unclassified_f_Lachnospiraceae*, *Bacteroides*, *Phascolarctobacterium*, *Escherichia-Shigella*, *[Ruminococcus]_torques_group*, *unclassified_f_Ruminococcaceae*, *Barnesiella*, *Faecalibacterium*, and *Olsenella* were the dominant genera, which were the top 10 genera in abundance values. Broilers in the antibiotics group had lower genus *unclassified_ f_Lachnospiraceae* than broilers in the control group (*p* < 0.05). The increased genus *Bacteroides*, and reduced genus *unclassified_f_Lachnospiraceae* and *Christensenellaceae_R-7_group* were observed in broilers fed the RLS addition diet compared with the control group (*p* < 0.05). Based on the Greengenes data, PICRUSt analysis showed that the known functional genes for immune disease and digestive system exhibited higher heatmap scores in birds fed the RLS-supplemented diet than those birds fed the control diet or antibiotics-supplemented diet (**Figure 6**).

### Cecal Volatile Fatty Acids

**Figure 7:**
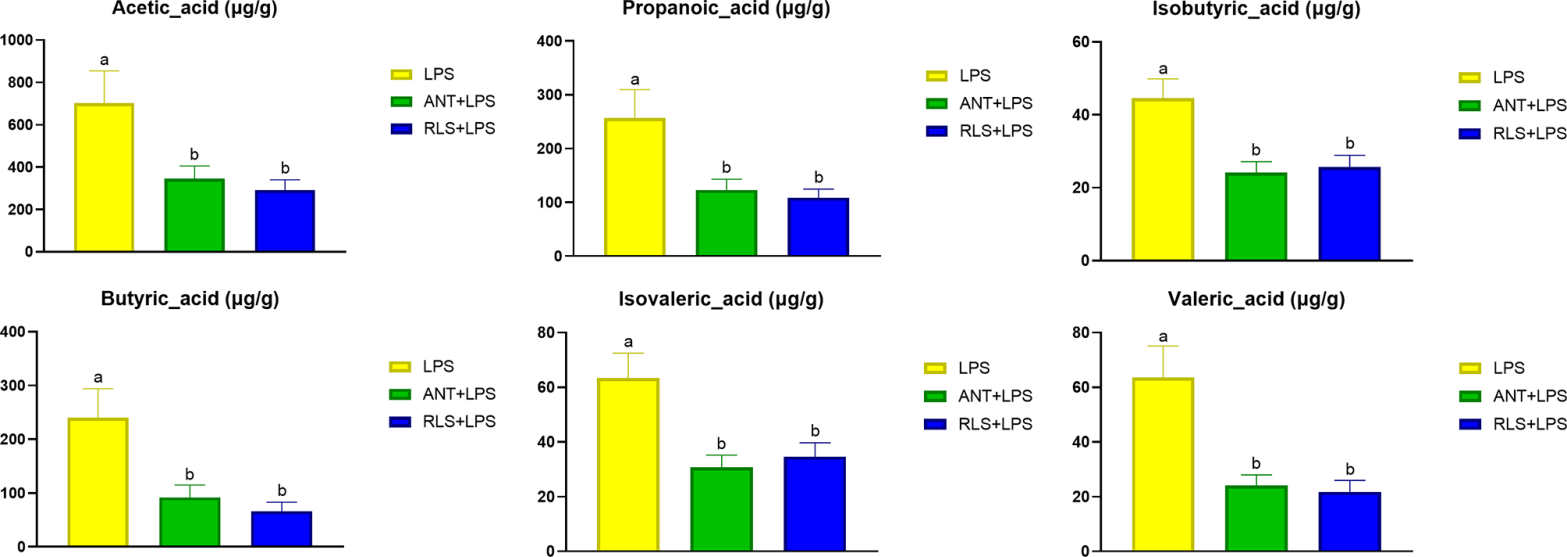
Rhamnolipids modulated cecal volatile fatty acids levels in broilers challenged with lipopolysaccharide (^ab^means that do not share the same superscript are significantly different, p < 0.05; *p < 0.05).

**Figure 8:**
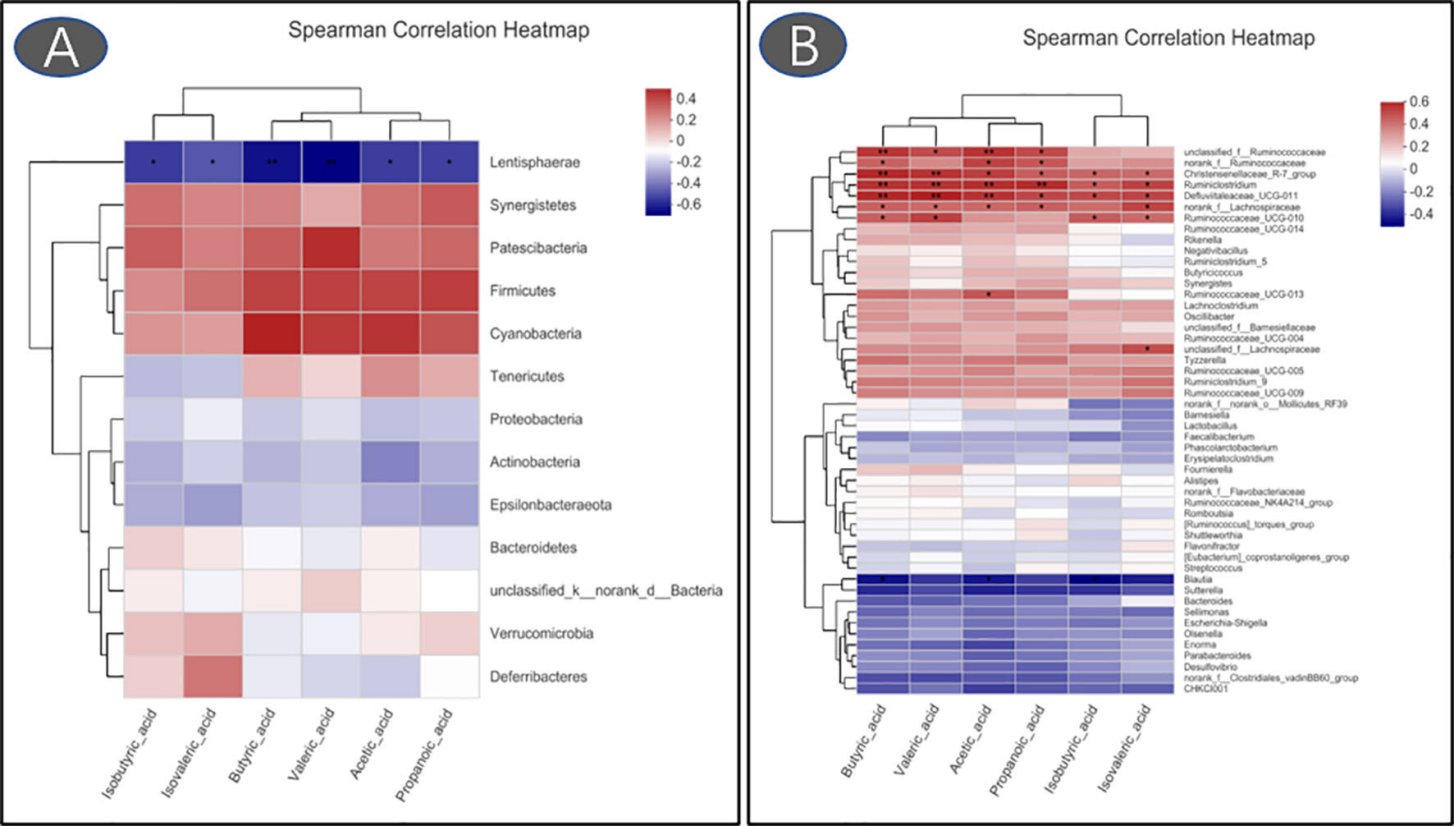
Heatmaps of Spearman’s correlation analysis between gut microbiota and volatile fatty acids (**A**: phylum; **B**: genus. *p < 0.05, **p < 0.01).

Compared with the control group, the reduced levels of acetic acid, propanoic acid, isobutyric acid, butyric acid, isovaleric acid, and valeric acid were observed in LPS-challenged broilers that were fed antibiotics or RLS-supplemented diet (*p* < 0.05, **Figure 7**). Additionally, Spearman’s correlation analysis showed that the phylum Lentisphaerae showed a significant negative correlation with acetic acid, propanoic acid, isobutyric acid, butyric acid, isovaleric acid, and valeric acid (*p* < 0.05, **Figure 8**). The genera *Christensenellaceae_R-7_group*, *Ruminiclostridium*, *Defluviitaleaceae_UCG-011*, and *norank_f_Lachnospiraceae* had significant positive correlation with acetic acid, propanoic acid, isobutyric acid, butyric acid, and valeric acid (*p* < 0.05).

### Metabolome Composition

**Figure 9:**
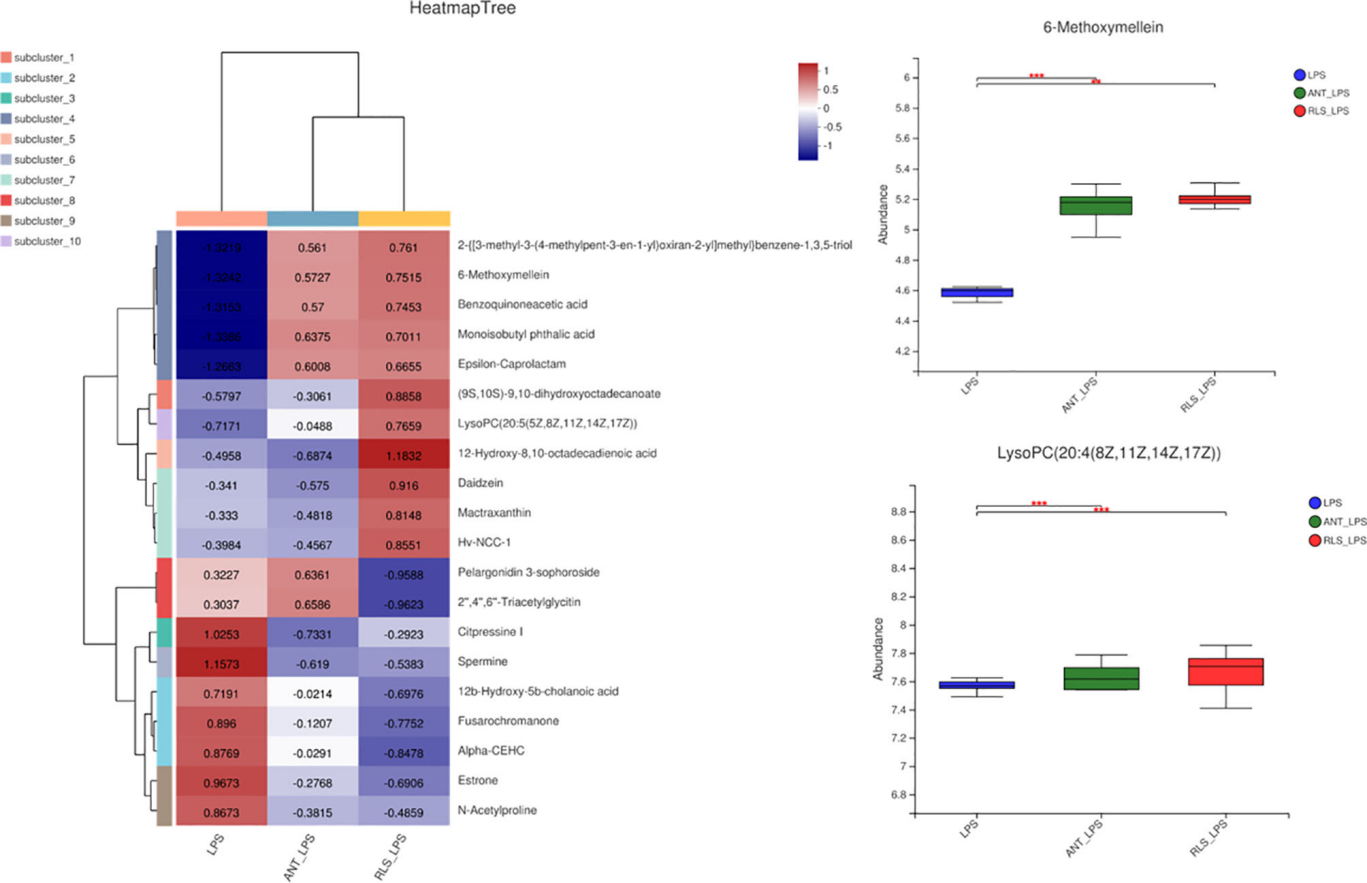
Rhamnolipids modulated the serum metabolome profile in broilers challenged with lipopolysaccharide (**p < 0.01, ***p < 0.001).

**Figure 10:**
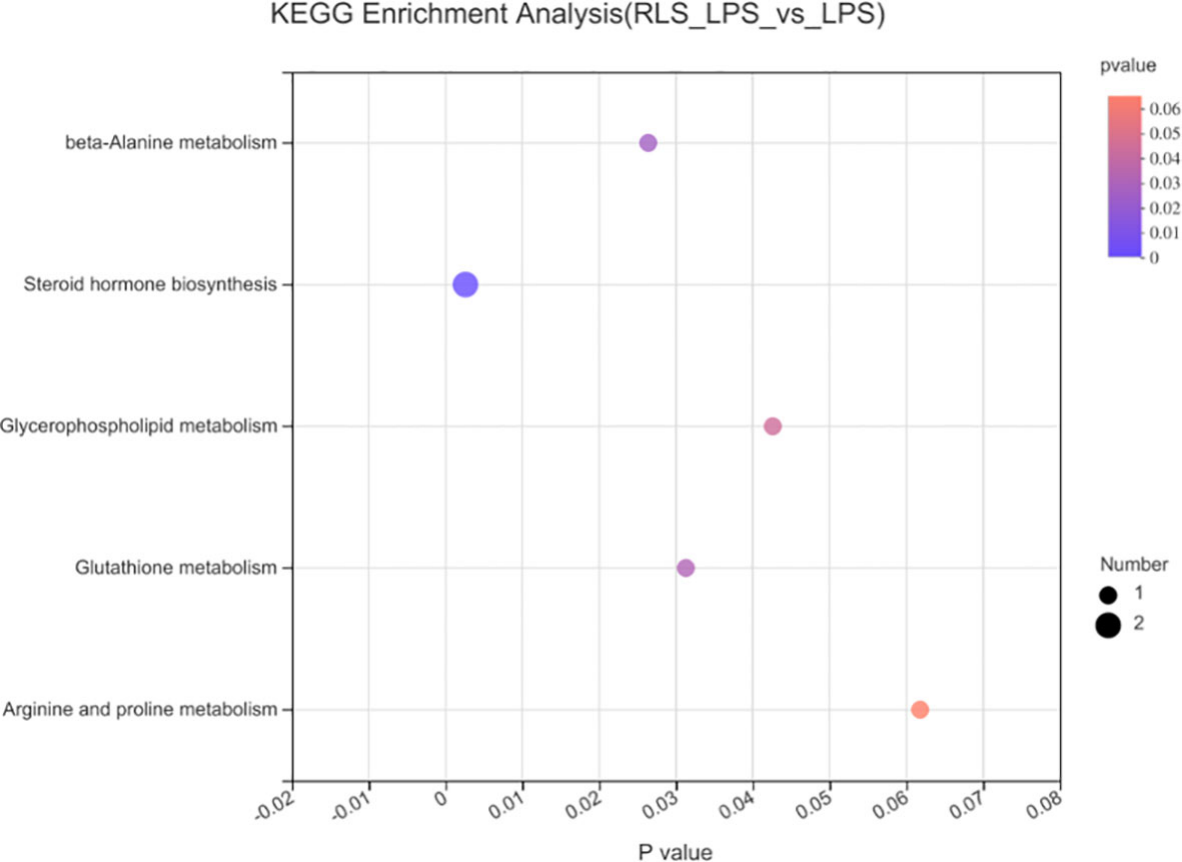
Metabolome view map of common metabolites identified in serum of broilers.

The heatmap trees showed that the metabolome profile of serum in broilers exhibited clear differences among the groups (**Figure 9**). RLS and antibiotic inclusion markedly upregulated the abundance of 6-methoxymellein and lysoPC [20:5(5Z,8Z,11Z,14Z,17Z)] in serum of LPS-challenged broilers (*p* < 0.05). The KEGG annotation enriched pathways were steroid hormone biosynthesis, glutathione metabolism, glycerophospholipid metabolism, arginine and proline metabolism, and beta-alanine metabolism in comparison to the RLS group and control group (**Figure 10**).

**Figure 11:**
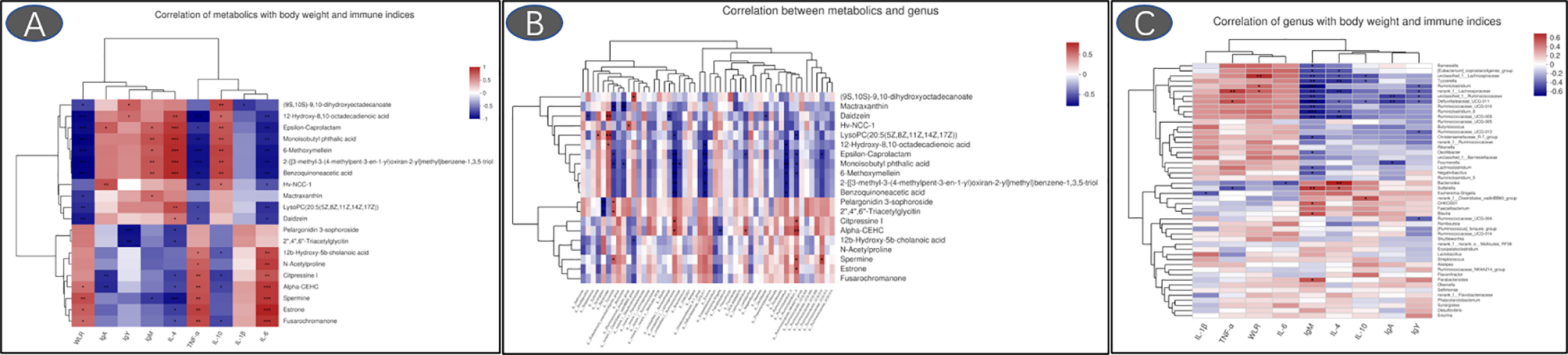
Heatmaps of Spearman’s correlation analysis (**A**: correlation of metabolics with body weight and immune indices; **B**: correlation between metabolics and genus; **C**: correlation of genus with body weight and immune indices; *p < 0.05, **p < 0.01, ***p < 0.001).

The 6-methoxymellein negatively correlated with the relative abundance of genus *Lachnospiraceae*, *Defluviitaleaceae_UCG-011*, and *Ruminiclostridium* (*p* < 0.05, **Figure 11**). A positive correlation was observed when comparing the 6-methoxymellein to IgM, IL-4, and IL-10 (*p* < 0.05). Negative correlations were observed when comparing the 6-methoxymellein to WLR, TNF-α, and IL-6 (*p* < 0.05). The serum metabolites lysoPC (20:5(5Z,8Z,11Z,14Z,17Z)) are positively correlated with the relative abundance of genus *Bacteroides* (*p* < 0.05) and serum IL-4 level, and negatively correlated with WLR, TNF-α, and IL-6 (*p* < 0.05). The genus *Bacteroides* also showed a significant negative correlation with IL-6, and had a positive correlation with serum IL-4 (*p* < 0.05).

## Discussion

Numerous studies have proven that broilers under LPS challenge exhibit compromised growth performance as evidenced by reducing feed intake, weight gain, and feed conversion ratio; disrupted intestinal barrier function; and the negotiation of nutrients away from growth to support various immunization-related processes, such as the synthesis of cytokines and various acute proteins (14, 20, 21). This research confirmed that LPS challenge decreased the body weight of broilers. Accumulating lines of evidence have demonstrated that RLS addition in diet could enhance the growth performance of birds (9, 10). The present study found that dietary antibiotics or RLS supplementation improved the weight loss rate induced by LPS challenge. The body weight and weight loss rate of broilers displayed no difference between the antibiotics group and the RLS group. These findings suggested that RLS may serve as an effective alternative to antibiotics and exert a positively protective effect on the growth of birds under the condition of immunological stress.

The immune system protects the body against foreign substances and guards invasion by pathogenic organisms (22). In chicks, immunoglobulins including IgA, IgY, and IgM are the important indicators to evaluate the status of the immune system (23). Significant reduced levels of serum IgA, IgY, and IgM were observed in LPS-challenged broilers. Moreover, broilers in the RLS group and ANT group exhibited higher serum IgA, IgY, and IgM contents than broilers in the LPS group. In agreement with this result, RLS supplementation increased the serum IgA, IgM, and IgY levels in Linnan yellow broilers (9). Cytokine secretion is normally vital for triggering the innate defense program and then modulating the immune response of an adaptive system (24). LPS can activate TLR 4-mediated inflammatory response pathways such as NF-κB to lure the generation of cytokines (25). NF-κB is a central regulator of various gene signaling involved in the innate immune response system (26). TNF-α, IL-6, and IL-1β are the potent proinflammatory and immunomodulatory cytokines (27), whereas IL-4 and IL-10 have been accepted as anti-inflammatory and tolerogenic cytokines that reduce the generation of proinflammatory cytokines (28). This research found that LPS challenge decreased the IL-4 and IL-10 contents, and increased the IL-6, IL-1β, and TNF-α levels in serum of broilers. These results were accompanied by the significant growth impairment and changes in serum immunoglobulin levels. The LPS-challenged induced production of these inflammatory cytokines of broilers was suppressed by RLS and antibiotic inclusion in the diet. In brief, RLS is effective for mitigating the inflammatory responses of broilers induced by LPS. Broilers in the RLS group had higher serum IL-4 and IL-10 contents and lower serum IL-6 and TNF-α levels than broilers in the antibiotic group, implying that RLS had better ability to alleviate the injury of LPS-challenged broilers than the antibiotic in the diet. The intestinal dysfunction was observed in LPS-challenged broilers (29). Presently, we found that LPS challenge upregulated the jejunum mucosa *TLR 4*, *NF-κB*, and *TNF-α* expression levels, and ileum mucosa *TLR 4*, *MyD88*, and *TNF-α* expression levels, and downregulated the expression level of jejunum and ileum mucosa *IL-4* in broilers. Dietary RLS inclusion decreased *TLR 4*, *NF-κB*, and *TNF-α* expression level, and increased *IL-4* expression level in jejunum mucosa of broilers. Similarly, RLS addition in the diet reduced the *MyD88* and *TNF-α* expression levels, and enhanced the *IL-4* expression level in ileum mucosa in LPS-challenged broilers. The results of this study reveal that LPS-induced inflammatory responses could be ameliorated by RLS *via* regulating the suppression of inflammatory cytokine synthesis. It is worth noting that dietary antibiotic supplementation only decreased the expression levels of jejunum mucosa *TLR 4* and *NF-κB*, and enhanced the jejunum and ileum *IL-4* expression levels in LPS-challenged broilers. The mucosa *TNF-α* expression levels exhibited no difference between the LPS-challenged group and the antibiotic group. In view of these results, we speculate that RLS may be more efficient than antibiotic in the diet of broilers to attenuate LPS-induced inflammatory responses.

The intestinal morphology can reveal the intestinal health status in animals (30). LPS challenge could cause intestinal dysfunction in broilers (29). This study found that broilers in the LPS group displayed obvious bleeding point in jejunum and ileum. RLS and antibiotics supplementation could improve the symptoms of intestinal bleeding in LPS-challenged broilers, implying that RLS could serve as an effective alternative to antibiotics attenuated LPS-induced intestinal injury in broilers. Furthermore, the levels of the serum D-LA and DAO could act as biomarkers of intestinal barrier integrity (31). The serum D-LA and DAO concentrations are enhanced in birds when the intestinal barrier is injured (32). The enhanced serum D-LA and DAO contents were observed in LPS-challenged broilers. RLS supplementation in diet reduced serum D-LA and DAO concentrations in LPS-challenged broilers. These findings indicated that RLS could abate intestinal permeability and lessen the degree of intestinal injury in broilers. Tight junctions, such as occludin, claudins, ZO-1, and MUC-2, are crucial components of the intestinal mucosal barrier, sustaining paracellular permeability (33). The present study found that the reduced jejunum *MUC-2*, *clandin-1*, and *zonula occludens-1* mRNA expression levels were improved by RLS and antibiotics, which is in keeping with the serum DAO and D-LA concentrations of broilers. This study showed that it had no significant differences in serum D-LA and DAO concentrations, and intestinal mucosal *occludin*, *claudins*, *ZO-1*, and *MUC-2* expression levels between the LPS group and antibiotics group, implying that RLS may serve as a good alternative to antibiotics supplementation in the diet of birds to alleviate the harmful consequences of LPS challenge on intestinal mucosal permeability, which is in keeping with the state of intestinal health in broilers.

The villus height, crypt depth, and the ratio of villus height to crypt depth of intestine are the common criteria for evaluating the nutrient absorptive property, and higher villus height means a higher absorptive capacity of the small intestine (34). LPS challenge reduced the villus height and decreased the ratio of villus height to crypt depth of jejunum and ileum in broilers. RLS could alleviate compromised intestinal morphology challenged by LPS in broilers through increasing villus height and decreasing the crypt depth in this study, indicating that RLS had a positive protective effect in LPS-challenged broilers. This is consistent with the body weight of broilers in this study.

Intestinal microbiota is vital for maintaining intestinal health and promoting growth, and has a vital role in resisting pathogenic infection in broilers (35). The Shannon index is used to evaluate the microbiota diversity of the sample (36). In the present study, RLS reduced the Shannon index of the cecal microbiota, suggesting that the microbial diversity of broilers in the RLS group is lower than that of broilers in the control group. A previous study found that RLS has various biomedical properties such as antimicrobial, anti-inflammatory, immunomodulator, and cellular differentiation agents (37). The positive antimicrobial activity of RLS may provide selective pressure to bacteria in broilers (38). The clear separations of PCoA also confirmed the differentiation of intestinal microflora in broilers between the control group and thew RLS group.

Phyla Firmicutes and Bacteroidetes are the predominant bacterial strains in this study, which is similar to previous studies (9, 10, 39). Studies demonstrated that Firmicutes and Bacteroidetes are linked to the efficiency of energy harvesting in poultry (40). Li et al. (41) reported that the growth of broilers had a positive relationship with the Bacteroidetes abundance and a negative relationship with the Firmicutes abundance. Here, LPS-challenged broilers in the RLS group increased the abundance of Bacteroidetes, and reduced the abundance of Firmicutes in cecal chyme. Previously, researchers confirmed that a variety of members in Bacteroidetes provide a beneficial role in host digestion in broilers (42). The PICRUSt analysis also showed that the known functional genes for the digestive system exhibited higher heatmap scores in broilers fed the RLS-supplemented diet than those birds fed the control diet or antibiotics-supplemented diet. These results implied that RLS may improve the function of digestion and absorption by regulating the intestinal Bacteroidetes. In this study, dietary supplementation with the antibiotics and RLS decreased the levels of acetic acid, propanoic acid, isobutyric acid, butyric acid, isovaleric acid, and valeric acid. Whether volatile fatty acids are reduced due to the increased metabolic function of intestinal epithelial cells needs to be elucidated, and further study needs to explore the specific mechanism. In addition, a study demonstrated that Firmicutes mainly produce butyrate, whereas Bacteroidetes produce acetate and propionate. The reduced abundance of Firmicutes and Bacteroidetes might illustrate the downregulation of VFA production of broilers in the RLS group and ANT group. Additionally, the present study showed that the phylum Lentisphaerae is negatively correlated with the levels of acetic acid, propanoic acid, isobutyric acid, butyric acid, isovaleric acid, and valeric acid in intestinal contents of broilers. However, there is little knowledge about the nutritional characteristics of Lentisphaerae. Whether Lentisphaerae can digest these short-chain fatty acids to synthesize its own metabolites or other mechanisms needs further study.

The genus *Bacteroides* exhibits a positive effect on host health and disease resistance, and has attracted widespread attention (43). The reduced abundance of *Bacteroides* was observed in patients with inflammatory bowel diseases (44–46). In this study, the cecal *Bacteroides* of broilers in the RLS group were substantially enhanced compared with the control and antibiotics group. Consistently, oral *Bacteroides fragilis* and *Bacteroides ovatus* relieved the inflammatory response induced by LPS through improving the intestinal microbiota, modulating the cytokine production, and maintaining the regulatory T cell (Treg)/Th-17 balance in mice (47). These results indicated that RLS may alleviate LPS-induced inflammatory responses *via* improving the genus *Bacteroides* abundance in broilers. Studies reveal that the genus *unclassified_f_Lachnospiraceae* is the main acetate-producing bacteria (48). The present study found that broilers in the RLS group had lower abundance of *unclassified_f_Lachnospiraceae* than broilers in the control group. Spearman’s correlation analysis also exhibited a significant positive correlation between acetic acid and the genus *unclassified_f_Lachnospiraceae*, which is mutually supporting with the acetic acid concentration in this study.

Serum metabolome profiles are important markers to monitor health, to diagnose and cure diseases, and to reflect the nutritional status of broilers. 6-methoxymellein is a subgroup of 3,4-dihydroisocoumarins, which belongs to the mellein family (49). This study found that RLS and antibiotics enhanced serum 6-methoxymellein levels. Spearman’s correlation analysis revealed that 6-methoxymellein negatively correlated with WLR, TNF-α, and IL-6, and positively correlated with IgM, IL-4, and IL-10. Previously, the inhibited nuclear localization of nuclear factor-κB subunit p65 and p50 was observed by 6-methoxymellein treatment in breast cancer stem cells. Subsequently, 6-methoxymellein reduced the expression of IL-6 (50). Collectively, these results suggest that RLS may alleviate LPS-induced inflammatory responses through altering the 6-methoxymellein level in broilers. LysoPCs are a class of lipid biomolecules and have a direct role in ameliorating systemic inflammatory disorders (51). RLS markedly enhanced the serum lysoPC(20:5(5Z,8Z,11Z,14Z,17Z)) level of broilers in this study. LysoPC(20:5(5Z,8Z,11Z,14Z,17Z)) also had a positive correlation with serum IL-4 and cecal genus *Bacteroides*, and exhibited negative correlation with IL-6. Moreover, the genus *Bacteroides* showed a significant negative correlation with IL-6 and had a positive correlation with serum IL-4. Thus, we speculate that rhamnolipids may alleviate the LPS-induced inflammatory responses *via* modulating the cecal genus *Bacteroides* and henceforth improving lysoPC (20:5(5Z,8Z,11Z,14Z,17Z)) secretion.

## Conclusion

Rhamnolipids can alleviate LPS-induced inflammatory responses and intestinal injury, and improve growth by enhancing immunity and ameliorating the intestinal microflora and the key functional serum metabolites in LPS-challenged broilers. The genus *Bacteroides* may contribute to the decreased WLR and enhanced immunoglobulins *via* regulating the serum lysoPC (20:5(5Z,8Z,11Z,14Z,17Z)) secretion. Rhamnolipids exhibited better protective effects than antibiotic supplementation in the diet of LPS-challenged broilers. These findings provide novel insights and potential regulation strategies for RLS enhancing its protective effect in LPS-challenged broilers.

## Data Availability Statement

The datasets presented in this study can be found in online repositories. The name of the repository and accession number can be found below: National Center for Bitechnology Information (NCBI) BioProject, https://www.ncbi.nlm.nih.gov/bioproject/, PRJNA785797. The other raw data supporting the conclusions of this article will be made available by the authors, without undue reservation.

## Ethics Statement

The animal study was reviewed and approved by Zhejiang Agricultural and Forestry University Animal Care and Use Committee.

## Author Contributions

RZ, XS, and YX conceived the project and designed the protocol. RZ, XS, YC, JL, YW, and YX performed the experiments. RZ, XS, and YX wrote the manuscript. All authors contributed to the article and approved the submitted version.

## Funding

The work was supported by the Zhejiang Provincial Leading Innovation and Entrepreneurship Team Project (2020R01015) and the Project of Key Agricultural Research Institute of Green Animal Health Products of Zhejiang Province (2021Y30004).

## Conflict of Interest

Authors YC and JL are employed by Zhejiang Vegamax Biotechnology Co., Ltd.

The remaining authors declare that the research was conducted in the absence of any commercial or financial relationships that could be construed as a potential conflict of interest.

## Publisher’s Note

All claims expressed in this article are solely those of the authors and do not necessarily represent those of their affiliated organizations, or those of the publisher, the editors and the reviewers. Any product that may be evaluated in this article, or claim that may be made by its manufacturer, is not guaranteed or endorsed by the publisher.
