# Supplementary material for: Multi-Omics Revealed the Protective Effects of Rhamnolipids in Lipopolysaccharide Challenged Broilers
Source: Front Immunol. 2022 Feb 18;13:824664. doi: 10.3389/fimmu.2022.824664 (PMC8895253; doi:10.3389/fimmu.2022.824664)
Supplement: Supplementary file 1 [file Table_1.docx]

Table S1 Composition and nutrient levels of basal diets (air-dry basis, %)

| Items | Contents | |
| --- | --- | --- |
|  | 1 to 21 days of age | 22 to 42 days of age |
| Ingredients |  |  |
| Corn | 52.5 | 54 |
| Soybean meal | 25 | 17 |
| Extruded soybean | 4.5 | 3.5 |
| DDGS | 8.5 | 7.5 |
| Rice bran | 0 | 6 |
| Corn gluten | 0 | 2 |
| Soybean oil | 1.7 | 4.6 |
| Limestone | 1.4 | 1.4 |
| Fermented soybean meal | 2.4 | 0 |
| Premix ^1^ | 4 | 4 |
| Total | 100.00 | 100.00 |
| Nutrient levels |  |  |
| CP | 22.02 | 19.11 |
| ME（MJ/kg） | 12.23 | 12.91 |
| EE | 5.5 | 8.6 |
| Lys | 1.18 | 0.97 |
| Met | 0.54 | 0.45 |
| Met+Cys | 0.88 | 0.74 |
| Thr | 0.86 | 0.71 |
| [Try](javascript:;) | 0.23 | 0.20 |
| Ca | 0.82 | 0.73 |
| TP | 0.65 | 0.57 |

^1^ The premix provided the following per kg of diets: V_A_ 10 000 IU, V_B1_ 2.2 mg, V_B2_ 8.0 mg, V_B5_ 40 mg, V_B6_ 4.0 mg, VB_12_ 0.013 mg, V_D3_ 3 000 IU, V_E_ 30 IU, V_K3_ 1.3 mg, biotin 0.04 mg, folic acid 40 mg, D-pantothenate calcium 10 mg, nicotinic acid 40 mg, choline chloride 400 mg, Cu 7.5 mg, Fe 80 mg, Mn 110 mg, Zn 65 mg, I 1.1 mg, Se 0.3 mg.

Table S2 Primer sequences for real-time PCR assays

| Gene^1^ | Genbank id | Primer sequence, sense/antisense | Product size, bp |
| --- | --- | --- | --- |
| *β-actin* | NM_205518.1 | TGCTGTGTTCCCATCTATCG  TTGGTGACAATACCGTGTTCA | 150 |
| *TLR2* | AB046533 | CATTCACCATGAGGCAGGGATAG  GGTGCAGATCAAGGACACTAGGA | 157 |
| *TLR4* | NM_001030693.1 | TGACCTACCCATCGGACACT  CTCAGGGCATCAAGGTCTCC | 171 |
| *MyD88* | NM_001030962.4 | GATGATCCGTATGGGCATGGA  ATGGACCACACACACGTTCC | 170 |
| *NF-κB* | XM_015285418.2 | TGCCTTTTGCTTGAGGGTGATG  CTGCCAGTTTTGTGAAGCCC | 100 |
| *IL-1β* | NM_204524.1 | GTACCGAGTACAACCCCTGC  AGCAACGGGACGGTAATGAA | 112 |
| *TNF-α* | NM204267.1 | CCGTAGTGCTGTTCTATGACCG  GTTCCACATCTTTCAGAGCATCAA | 235 |
| *Mucin 2* | XM_001234581.3 | AGGAATGGGCTGCAAGAGAC  GTGACATCAGGGCACACAGA | 77 |
| *Occludin* | NM_205128.1 | AGCCCTCAATACCAGGATGTG  CGCTTGATGTGGAAGAGCTTG | 125 |
| *Claudin-1* | NM_001013611.2 | CACACCCGTTAACACCAGATTT  GAGGGGGCATTTTTGGGGTA | 159 |
| *zonula occludens-1* | XM_015278980.2 | GGATGTTTATTTGGGCGGCT  CCATTGTTGCACTCTTGCCG | 153 |

^1^ *TLR2, 4*=toll-like receptors 2, 4; MyD88=myeloid differentiation factor 88; *NF-κB*=nuclear factor-kappa B; *IL-1β*=interleukin-1β; *TNF-α*=tumor necrosis factor-α; *ZO-1*=zonula occludens-1.
